# Preparation of Three-Dimensional Vascularized MSC Cell Sheet Constructs for Tissue Regeneration

**DOI:** 10.1155/2014/301279

**Published:** 2014-07-08

**Authors:** Liling Ren, Dongyang Ma, Bin Liu, Jinda Li, Jia Chen, Dan Yang, Peng Gao

**Affiliations:** ^1^School of Stomatology, Lanzhou University, Lanzhou, Gansu 730000, China; ^2^Department of Oral and Maxillofacial Surgery, Lanzhou General Hospital, Lanzhou Command of PLA, Gansu 730050, China

## Abstract

Engineering three-dimensional (3D) vascularized constructs remains a challenge due to the inability to form rich microvessel networks. In this study we engineered a prevascularized 3D cell sheet construct for tissue regeneration using human bone marrow-derived mesenchymal stem cells (hMSCs) and human umbilical vein endothelial cells as cell sources. hMSCs were cultured to form a thick cell sheet, and human umbilical vein endothelial cells (HUVECs) were then seeded on the hMSCs sheet to form networks. The single prevascularized HUVEC/hMSC cell sheet was folded to form a 3D construct by a modified cell sheet engineering technique. *In vitro* results indicated that the hMSCs cell sheet promoted the HUVECs cell migration to form networks in horizontal and vertical directions. *In vivo* results showed that many blood vessels grew into the 3D HUVEC/hMSC cell sheet constructs after implanted in the subcutaneous pocket of immunodeficient mice. The density of blood vessels in the prevascularized constructs was higher than that in the nonprevascularized constructs. Immunohistochemistry staining further showed that *in vitro* preformed human capillaries in the prevascularized constructs anastomosed with the host vasculature to form functional blood vessels. These results suggest the promising potential of this 3D prevascularized construct using hMSCs cell sheet as a platform for wide applications in engineering vascularized tissues.

## 1. Introduction

Synthetic tissue engineering scaffolds including bioceramic, polymer, or composite scaffolds have been extensively studied for the application of tissue regeneration due to their excellent biocompatibility [1, 2]. However, the success in using these synthetic scaffolds to regenerate tissue remains limited, especially in the regeneration of thick tissues like heart, kidney, or bone [3, 4]. One of the main reasons that results in the failure of implantation is insufficient vascularization in constructs after implantation [5]. Limited nutrient diffusion and slow growth of new vessels often cause necrosis at the core in the large constructs [6, 7]. A large number of methods have been developed to improve the vascularization of tissue engineering constructs and have achieved some degree of success. These approaches mainly include delivering growth factors and cytokines [8], culturing endothelial cells on the synthetic constructs [9], and coculturing endothelial progenitor cells and pericytes [10]. However, the vascularization of the synthetic constructs remains insufficient for efficient formation of functional blood vessels. This limited vascularization ability of the synthetic constructs mainly results from the lack of an extracellular matrix (ECM) microenvironment on synthetic scaffolds [11]. Studies have shown that ECM plays a critical role in promoting endothelial cell to form vascularization [12, 13]. Therefore, new strategies are required to develop a vascularized tissue-engineered construct that contains a rich ECM for efficiently promoting the formation of a functional vessel system.

Scaffold-free cell sheet engineering technology has showed a promising potential to produce a rich and intact ECM with a high density of cells inside [14]. The technique uses a thermosensitive culture surface to detach a confluent cell sheet [15, 16], thus engineering a specific tissue [12, 13, 17]. Studies have showed that some vascularized engineering tissues, such as corneas [18], myocardium [19], esophagus [20], pancreas [21], blood vessel [22], skeletal muscle [23], and periodontal ligament [24], have been successfully fabricated by cell sheet engineering technology. However, using human bone marrow mesenchymal stem cells (hMSCs) as a cell source to develop cell sheet constructs and investigating the vascularization ability of endothelial cells on the hMSCs sheet have not been fully explored.

Studies have shown that MSCs can stabilize the newly formed blood vessels as a pericyte through direct cell-cell contact with endothelial cells [25], and studies also found that a perivascular MSC niche* in vivo* can aid the outgrowth of endothelial cells and promote the early sprouts* in vivo *[26]. Some surface markers or important adhesion molecules expressed by MSCs also play important roles for endothelial cell activity and angiogenesis* in vivo* and* in vitro *[27]. Therefore, using hMSCs as a cell source to produce a prevascularized cell sheet construct for tissue engineering applications will provide new potentials, not only because hMSCs have multipotent differentiation ability but also because hMSCs can functionally promote vascularization as a pericyte [18].

Therefore, the aims of this study are to develop a highly prevascularized 3D cell sheet construct through seeding HUVECs on an hMSCs cell sheet and to investigate its vascularization potential* in vivo.* We hypothesized that this prevascularized 3D cell sheet construct can promote the formation of blood vessels and functional anastomosis with host vasculature. In this study* in vitro* cell migration and network formation were investigated, and histological examinations were performed to evaluate the* in vivo* vascularization ability.

## 2. Materials and Methods 

### 2.1. Cell Culture

The hMSCs were purchased from Lonza (Shanghai, China) and cultured in a Dulbecco's Modified Eagle media (DMEM, Invitrogen, USA) with 10% fetal bovine serum (FBS), 1% antibiotic-antimycotic solution (contains 10,000 units/mL of penicillin, 10,000 *μ*g/mL of streptomycin, and 25 *μ*g/mL of Fungizone) under standard conditions (5% CO_2_, 95% humidity, and 37°C). Green fluorescent protein expressing HUVECs were purchased from Angio-Proteomie (Boston, USA) and cultured in EBM (endothelial basal medium) and an EGM (endothelial growth media) SingleQuots Kit. The cell medium was changed every 3 days. The passage of the two cells used in all the experiments was below 7.

### 2.2. Preparation and Characterization of Prevascularized HUVEC/hMSC Cell Sheet

**Figure 1:**
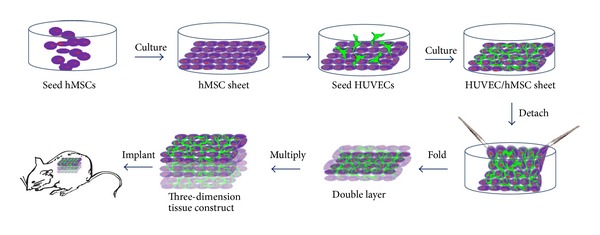
The schematic describes the procedures of preparing a three-dimensional prevascularized construct based on a modified cell sheet engineering technique. A cell scraper was used to scratch the cell sheet and a point forceps was then used to fold the cell sheet 3 times to form a square-like construct with 8 layers. The width was approximately 8 mm and length was around 8 mm.

hMSCs were first seeded on a cell culture dish at a cell density of 9 × 10^4^/cm^2^ and cultured in the basal culture DMEM medium (Figure 1). After 2 days, hMSCs reached confluence, and the DMEM medium was changed to DMEM cocktail containing 50 *μ*g/mL ascorbic acid and 30 mM glucose to promote the production of extracellular matrix [28]. After 14 days, hMSCs formed a thick cell sheet. HUVECs were then seeded onto the hMSCs sheet at a cell density of 5 × 10^4^/cm^2^. A fluorescent microscope was used to observe the HUVECs cell migration on the hMSCs sheet. At the same time, HUVECs were seeded on a tissue culture plate as a control. After 7 days, the HUVEC/hMSC cell sheet was fixed in 4% paraformaldehyde for 15 minutes, and immunofluorescent staining was performed to observe the network formation of HUVECs on the hMSCs sheet. A specific marker of HUVECs, platelet endothelial cell adhesion molecule (PECAM-1 or CD31), was used to assess the networks structure of HUVECs. After washing the HUVEC/hMSC sheet three times in PBS, the cell sheet samples were blocked in a 5% goat serum-PBS buffer solution for 1 hour at room temperature. A primary antibody rabbit anti-human CD31 (ab76533, Abcam, dilution 1 : 500) in 1% BSA-PBS was added into the sample and incubated overnight at 4°C. Then a secondary antibody goat-anti-rabbit (Alexa Fluor 594, 2 *μ*g/mL, Invitrogen) in 1% BSA-PBS buffer was added and incubated in the dark for 1 hour at room temperature. A DAPI (5 *μ*g/mL) solution was added to counterstain the cell nuclei for 5 minutes and then the sample was washed using PBS. The fluorescent staining was recorded by a confocal microscopy (Olympus). To observe the prevascularized network structure inside the hMSCs sheet, the HUVEC/hMSC cell sheets were fixed in 4% paraformaldehyde solution for 15 minutes. 10% sucrose and 15% sucrose in PBS were added into the sample and incubated for 3 h each at room temperature, and then the cell sheet immersed in 20% sucrose in PBS overnight at 4°C [29]. The treated cell sheets were then frozen in Tissue-Tek O.C.T. compound. A cross section of the HUVEC/hMSC cell sheet was cryoprocessed with a thickness of 8 *μ*m. An immunofluorescent staining of CD31 was performed on the sections following the procedures described above.

### 2.3. Preparation of Three-Dimensional Prevascularized HUVEC/hMSC Constructs

A modified cell sheet folding technique was used to construct a 3D prevascularized HUVEC/hMSC construct in this study. After culturing HUVECs on the hMSCs sheet for 7 days in a 100 mm cell culture dish, a single thick HUVEC/hMSC cell sheet was formed. A strip with approximately 8 mm width and 80 mm length was cut. A cell scraper was used to gently scratch the edge of the thick cell sheet and detach it. A point forceps was then used to fold the HUVEC/hMSC sheet 3 times to form HUVEC/hMSC constructs with 8 layers (Figure 1). The HUVEC/hMSC construct was further cultured for 1 day to integrate the 8 layer cell sheets into one unit. The width of the construct was approximately 8 mm and length was around 8 mm. The cell sheet construct was fixed in 4% paraformaldehyde solution and prepared the same embedding procedures as described in Section 2.2. To observe the 3D network structure inside the cell sheet construct, an 8 *μ*m thick cross section was cryosectioned. The immunofluorescent staining of CD31 was then performed.

### 2.4. *In Vivo* Subcutaneous Implantation

The* in vivo* animal study was approved by the Animal Care Panel of Lanzhou University. The 3D prevascularized HUVEC/hMSC constructs were prepared as described in Figure 1. hMSCs constructs without HUVECs were prepared as a control according to the same procedures. To ensure the thickness of the two constructs to be the same, the times of folding of the HUVEC/hMSC and hMSCs cell sheets were the same to get the same layers of cell sheets. Afterwards, the formed 3D cell sheet constructs were incubated for 24 h in an endothelial cell medium (EBM-2) before implantation. The cell sheet constructs were implanted into subcutaneous dorsal pockets of immunodeficient mice. In this study at each time point for each group, 6 male BALB/c immunodeficient nude mice (7-week-old, 20–25 g body weight, Vital River Laboratories, Beijing, China) were implanted. The two cell sheet constructs (HUVEC/hMSC and hMSCs) were implanted into each mouse. Before and after surgery, 25 *μ*g cefazolin/g and 0.1 *μ*g buprenorphine/g were administrated, and one mouse per cage was housed. After 1 and 2 weeks, the constructs were removed for histological analysis and evaluation of vascularization.

### 2.5. H&E Staining and Immunohistochemistry Staining

At the end of time period, the mice were euthanized and the cell sheet implants were removed, fixed in 10% formalin buffer solution for 24 h, dehydrated in serial degraded ethanol (70%, 90%, 95%, and 100%), embedded in paraffin, and then sectioned. Conventional hematoxylin & eosin (H&E) staining was performed on the 8 *μ*m thick sections to observe the formation of luminal blood vessel structures containing red blood cells. For immunohistochemistry staining of human CD31, sections were deparaffinized and retrieved by an antigen retrieval solution at 95–100°C for 20 minutes, then the sections were washed by PBS three times and blocked by blocking serum (5%) for 30 minutes. Rabbit anti-human primary antibody CD31 (ab76533, Abcam, dilution 1 : 250) was used. Goat anti-rabbit biotinylated secondary antibody (1 : 500; Vector Laboratories) and DAB substrate kit (Vector Laboratories) were used followed by hematoxylin counterstaining and permanent mounting. Six random view fields (under 40x magnification) of H&E or CD31 staining slides from 6 individual mice were imaged to quantify the blood vessels or human derived lumens formed in the cell sheet constructs* in vivo*, respectively. The luminal structures containing erythrocytes were defined as blood vessels. The density of the blood vessels was reported as mean values ± the standard deviation. The density of human cell derived lumens containing murine erythrocyte in the constructs was also quantified according to the same method.

### 2.6. Statistical Analysis

A Student's* t*-test was performed to statistically analyze the difference between the two groups. If the *P* value was less than 0.05, the difference was considered significant.

## 3. Results

### 3.1. HUVECs Morphology on an hMSCs Sheet

**Figure 2:**
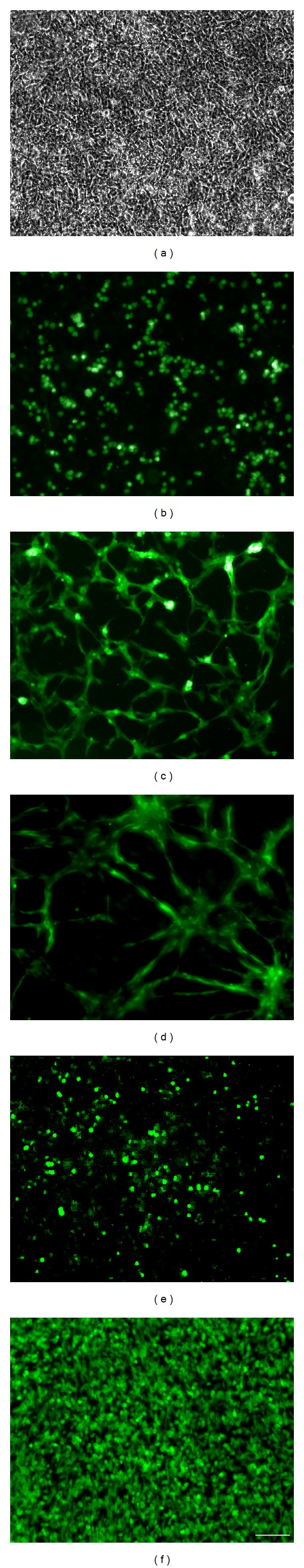
Images show the cell morphology of hMSCs after 14 days (a). It reached confluence and formed a thick cell layer. Fluorescent images ((b)–(d)) show the progressive process of HUVECs networks on an hMSCs sheet after HUVECs were seeded on the hMSCs sheet for 2 hours (b), 1 day (c), and 7 days (d). Fluorescent images ((e)-(f)) show the morphology of HUVECs seeded on tissue culture plate after 2 hours (e) and 7 days (f). HUVECs on the tissue culture plate proliferated but did not form networks. (Scale bar = 100 *μ*m).

To form an hMSCs sheet, we cultured hMSCs in an undifferentiated medium with a high concentration of glucose. After 14 days, hMSCs formed a dense cell sheet.  Figure 2(a) shows the dense morphology of the hMSCs sheet, which shows a rich ECM in the cell sheet. After obtaining a dense hMSCs sheet, we seeded HUVECs on the hMSCs sheet and cultured them in an endothelial medium. Once seeded, HUVECs adhered and started to migrate. After 2 hours, HUVECs showed spotty distribution on the hMSCs cell sheet (Figure 2(b)). After 1 day, the HUVECs migrated to connect with each other and formed networks with time increasing (Figure 2(c)). These networks continued to develop and formed longer branches at day 7 (Figure 2(d)). As a control, we seeded HUVECs on a tissue culture plate at the same time. Results show that HUVECs adhered to the well plate (Figure 2(e)) and proliferated to reach confluence, but they did not form any network during the culture time (Figure 2(f)).

### 3.2. Network Formation on the hMSCs Sheet

**Figure 3:**
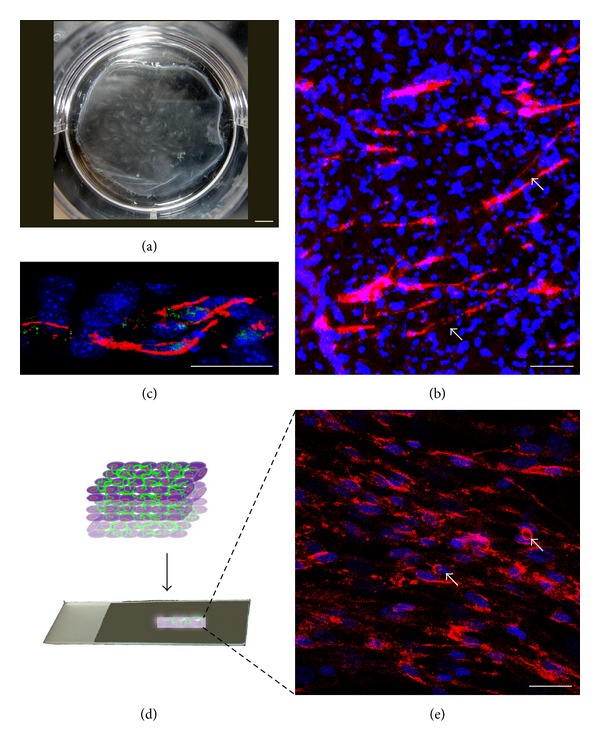
This picture shows the overall view of a cell sheet (a) (Scale bar = 3.4 mm). Confocal microscopy images show the lumens formation of HUVECs on hMSCs sheet after 7 days of culture. Immunofluorescent staining images of CD31 show lumens formed on the hMSCs sheet (b) (Scale bar = 100 *μ*m) and migrated into the sheet (c) (cross section view, Scale bar = 50 *μ*m). The three-dimensional prevascularized construct made by folding a single cell sheet was cryosectioned (d) and CD31 staining shows that networks and lumens formed in the construct (e) (white arrows, Scale bar = 100 *μ*m).

To investigate the network formation of HUVECs on the hMSCs sheet, immunofluorescent staining for CD31, a specific endothelial marker, was performed. After culturing HUVECs on the hMSCs sheet for 7 days, the cell sheet was fixed and stained. Immunofluorescent staining result shows that many networks formed on the hMSCs sheet (Figure 3(b)). From the image, it seems that the networks had vertically migrated into the inner portion of the hMSCs sheet not only just on the surface. To confirm this finding, we embedded the single layer of HUVEC/hMSC sheet (Figure 3(a)) into O.C.T. compound and cryosectioned it. The immunofluorescent staining on the cross section of the HUVEC/hMSC sheet shows that the network had migrated into the bottom of the hMSCs sheet and formed capillary lumens (Figure 3(c)). This result indicates that HUVECs formed networks inside the hMSCs sheet. From the cross section, it was seen that the thickness is around 30 *μ*m. We further folded the single layer of HUVEC/hMSC sheet (Figure 3(a)) into multiple layers of the cell sheet (Figure 3(d)) and cryosectioned it. Immunofluorescent staining for CD31 result shows that a large number of networks formed in the multiple layer cell sheets, and capillary and luminal structures were seen in the 3D cell sheet construct (Figure 3(e), white arrows). These confocal images show the trend of vertical migration of networks. At the same time, these lumens also demonstrated the formation of vascularized networks in the 3D thick cell sheet* in vitro*.

### 3.3. The* In Vivo* Formation of Functional Microvessels

**Figure 4:**
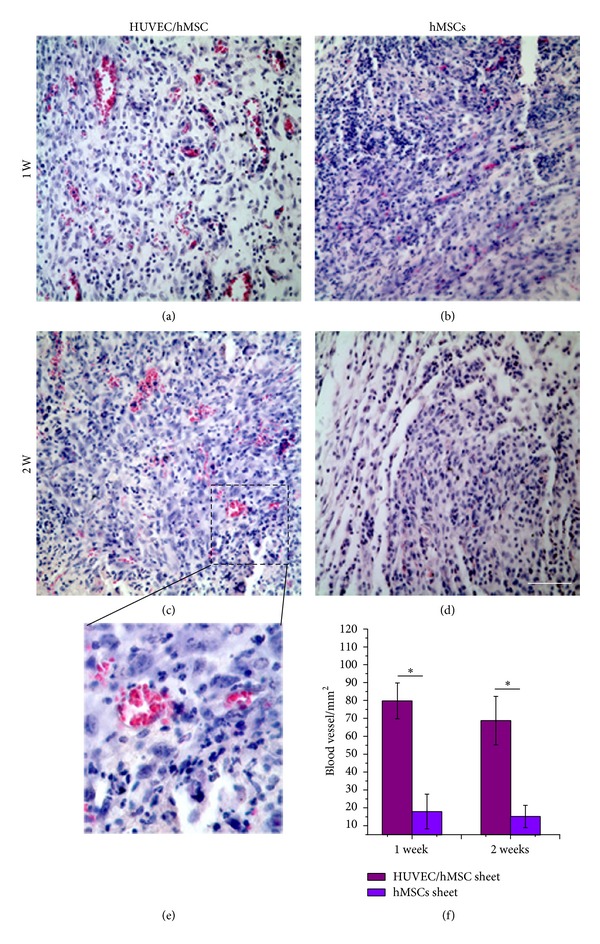
H&E staining of implants* in vivo*. H&E staining reveals that a large number of blood vessels grew into the HUVEC/hMSC constructs at 1 week (a) and 2 weeks (c), but few blood vessels were observed in hMSCs constructs ((b), (d)). A magnified image from (c) shows murine blood cells in a human derived blood vessel lumen (e). Blood vessel densities of the two groups at 1 and 2 weeks (f) (*P < 0.05, *n* = 6) (Scale bar = 100 *μ*m).

To examine whether the prevascularized networks can support the formation of functional vascular networks and rapidly anastomose with host vasculature, we folded the prevascularized HUVEC/hMSC sheet to prepare a 3D thick cell sheets construct and implanted the prevascularized constructs into subcutaneous pockets of immunodeficient mice. The undifferentiated hMSCs sheet without HUVECs served as a control. After 7 and 14 days after implantation, the constructs were removed. Hematoxylin & eosin (H&E) staining on paraffin sections showed that many blood vessels formed and were uniformly distributed in the HUVEC/hMSC sheet constructs (Figures 4(a) and 4(c)). A magnified image from Figure 4(c) shows the blood vessels containing erythrocytes (red; Figure 4(e)). In contrast, in the hMSCs sheet without HUVECs, there were few blood vessels observed. Quantitative results show that the density of the blood vessels in prevascularized HUVEC/hMSC sheet constructs is significantly higher than that in nonprevascularized hMSCs sheet constructs at days 7 and 14 (*P* < 0.05, *n* = 6) (Figure 4(f)). There is no significant difference in density of blood vessels of these two groups between the two implanted time points.

**Figure 5:**
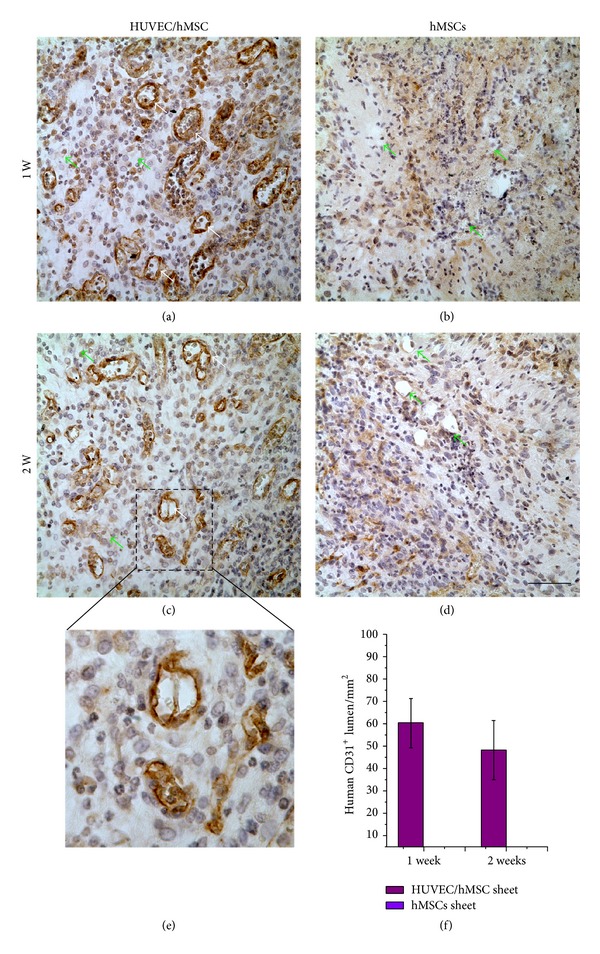
Immunohistochemistry staining images of CD31 for implants* in vivo*. Results show that human CD31 positive-expressing lumens (white arrows) and some invading host blood vessels (green arrows) were observed in HUVEC/hMSC constructs at 1 week (a) and 2 weeks (c), but there were only invading host blood vessels (green arrows) in the nonprevascularized group ((b), (d)). A magnified image from (c) shows that the human derived lumen (brown color) carried murine blood cells (e). Quantitative result shows the density of the human CD31 positive-expressing in the HUVEC/hMSC constructs at 1 and 2 weeks, but there is no significant difference between 1 and 2 weeks (f). (Scale bar = 100 *μ*m).

To further assess the functional anastomosis of preformed human vascular networks with the host vasculature* in vivo*, immunohistochemistry staining for human CD31 was performed on the paraffin sections. The vessels were stained positive for human CD31 confirming the implanted HUVECs networks, but lumens containing murine erythrocytes without human CD31 expression were thought to be murine blood vessels invading into the cell sheet constructs. The functional anastomosis was defined as a positive expression lumen containing murine erythrocytes, confirming that the implanted HUVECs networks were lined with murine blood vessels. Results showed that numerous blood vessels with positive expression of human CD31 were present in the prevascularized HUVEC/hMSC sheets (white arrows, Figures 5(a) and 5(c)). At the same time, some murine blood vessels invading into the constructs were seen (green arrows, Figures 5(a) and 5(c)). A magnified image from Figure 5(c) shows that positive human CD31 lumen contains murine blood cells (Figure 5(e)), which indicates that the* in vitro* prevascularized networks anastomosed with murine's vascular system and formed functionally perfused blood vessels. In contrast, few invading murine blood vessels were observed in the hMSCs sheets without HUVECs at day 7 or 14 (Figures 5(b) and 5(d)). Quantitative results show that there is no significant difference in the density of human lumens in the HUVEC/hMSC sheets between days 7 and 14 (Figure 5(f)).

## 4. Discussion

Rapid and sufficient vascularization in tissue-engineered constructs is a prerequisite for optimal cell survival and implants integration and has become one of the major challenges in thick tissue regeneration [6, 7]. In this study we found that an hMSCs cell sheet promoted network formation of HUVECs. HUVECs seeded on the hMSCs cell sheet formed rich networks, while on the tissue culture plate, no networks formed. Furthermore, the 3D HUVEC/hMSC constructs formed a large number of functional blood vessels* in vivo*, while the hMSCs sheet constructs without HUVECs formed few blood vessels. These findings suggest that the hMSCs cell sheets play a critical role in the network formation and vascularization process. ECM produced by hMSCs may provide proper microenvironments for HUVECs migration, proliferation, and network formation [30].

Our* in vitro* result further indicated that HUVECs migrated and formed networks on the hMSCs sheet not only in a horizontal direction but also in a vertical direction. This result implied that the hMSCs sheets provide a 3D environment for HUVECs growth and migration for network formation in all directions. Similar results can be found in Nagamori's study, which reported that HUVECs underneath a multilayered skeletal myoblast sheet actively and vertically migrated into the inner portions of the sheet [31]. The mechanism is still unknown. The possible reasons are that cell sheets provide rich ECM proteins and release various growth factors to initiate endothelial cell migration and networks sprouting through a paracrine pathway [32]. Endothelial cells may also rearrange and digest the ECM of cell sheets to create space for tube formation [30]. Although the mechanism remains to be investigated, we did find that 3D HUVECs networks formed on monolayer hMSCs sheets and in the 3D multiple cell sheet constructs. This structure may facilitate the rapid anastomosis* in vivo* with host vasculature. Meanwhile, a part of hMSCs possibly differentiated to endothelial-like cells under the culture condition, as we observed some positive expression of CD31 in Figures 5(b) and 5(d). This observation suggested that the culture medium may have effects on the differentiation of hMSCs. However, they did not form lumen structure.

In this study we modified the cell sheet technology based on Okano's cell sheet stacking method. In this process, we folded the single cell sheet to form a 3D construct without using thermoresponsive culture dishes and cell sheet stacking manipulation procedures [29]. The folding manipulation method in this study provides a potential to construct an arbitrary 3D construct, which can be flexibly fitted into irregular shapes of complex thick tissues, for example, an irregular bone tissue defect in craniomaxillofacial regions [33]. In this study we created a flexible 3D multiple layer construct that can promote rapid vascularization by this method, and the* in vivo* results confirmed that there were a large number of blood vessels which grew into the constructs and functionally anastomosed with the* in vitro* preformed human derived capillaries. Therefore, it is worth noting that although we did not use thermoresponsive culture dishes, this simple modified cell sheet technique can also detach intact cell sheets and form a multiple layer prevascularized 3D tissue construct.

Studies have indicated that MSCs have the capability of differentiating into multiple cell lineages, including osteocytes, chondrocytes, and adipocyte [34]. In addition, it has been reported that MSCs* in vitro* can support new vessel formation as pericytes [35] and can stimulate endothelial cell migration by releasing some angiogenic factors [36]. In this study we utilized a human MSC cell sheet and seeded HUVECs onto the cell sheet, which provides a vascularization microenvironment and also has stem cells sources for tissue differentiation. Our results confirmed the robust vascularization ability, but specific applications would need to be investigated, such as for bone regeneration.

Our main aim in this study is to investigate the vascularization ability of a 3D HUVEC/hMSC cell sheet construct. Our subcutaneous implantation results showed the formation of functional blood vessels in the constructs. However, the* in vivo* regeneration abilities of this cell sheet construct for specific tissues still need to be further investigated. In additional, more experiments need to be performed to investigate the mechanism of vascularization of HUVEC/hMSC cell sheet to fully understand the roles of cell sheet matrix components and for further improvement of vascularization.

## 5. Conclusion

In this study we developed a cell sheet using hMSCs as cell source and seeded HUVECs on the cell sheet to form a prevascularized cell sheet, which was further folded to fabricate 3D prevascularized cell sheet constructs. This construct showed remarkable vascularization ability* in vitro* and* in vivo*. Results suggested that the 3D prevascularized HUVEC/hMSC cell sheet construct provides a promising potential as a vascularization platform for wide applications in tissue regeneration.
